# Diaqua­dichloridomethyl­phenyl­tin(IV)–1,4,7,10,13-penta­oxacyclo­penta­decane (1/1)

**DOI:** 10.1107/S1600536812002693

**Published:** 2012-01-31

**Authors:** Marzieh Vafaee, Mostafa M. Amini, Seik Weng Ng

**Affiliations:** a Department of Chemistry, General Campus, Shahid Beheshti University, Tehran, Iran; b Department of Chemistry, University of Malaya, 50603 Kuala Lumpur, Malaysia, and Chemistry Department, Faculty of Science, King Abdulaziz University, PO Box 80203, Jeddah, Saudi Arabia

## Abstract

The asymmetric unit of the title cocrystal, [Sn(CH_3_)(C_6_H_5_)Cl_2_(H_2_O)_2_]·C_10_H_20_O_5_, contains two independent for­mula units. The organotin molecules exhibit a six-coordinate metal atom and are linked to the crown ether molecules by water–crown ether O—H⋯O hydrogen bonds into a linear chain running along [101]. Each coordinated water mol­ecule forms a pair of hydrogen bonds to the same crown ether; for the crown ether mol­ecules, only four of the five O atoms are engaged in hydrogen-bonding inter­actions. The metal ions show a distorted *trans*-C_2_SnCl_2_O_2_ octa­hedral coordination geometry [C—Sn—C = 175.3 (1) and 178.9 (1)°].

## Related literature

For a related compound, [MePhSnCl_2_(H_2_O)_2_]_2_·18-crown-6, see: Amini *et al.* (1994 ▶).
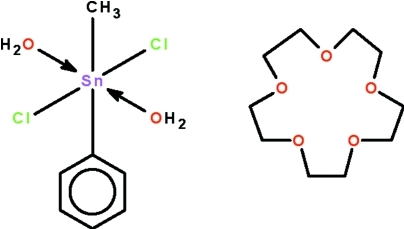


## Experimental

### 

#### Crystal data

[Sn(CH_3_)(C_6_H_5_)Cl_2_(H_2_O)_2_]·C_10_H_20_O_5_
*M* *_r_* = 538.02
Monoclinic, 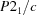

*a* = 16.4338 (3) Å
*b* = 21.2112 (4) Å
*c* = 14.0347 (3) Å
β = 113.325 (2)°
*V* = 4492.40 (15) Å^3^
*Z* = 8
Mo *K*α radiation
μ = 1.41 mm^−1^
*T* = 100 K
0.30 × 0.25 × 0.20 mm

#### Data collection

Agilent SuperNova Dual diffractometer with Atlas detector
Absorption correction: multi-scan (*CrysAlis PRO*; Agilent, 2011 ▶) *T*_min_ = 0.677, *T*_max_ = 0.766
39686 measured reflections
10200 independent reflections
8806 reflections with *I* > 2σ(*I*)
*R*_int_ = 0.038

#### Refinement

*R*[*F* ^2^ > 2σ(*F* ^2^)] = 0.028
*wR*(*F* ^2^) = 0.078
*S* = 1.05
10200 reflections
521 parameters
12 restraints
H atoms treated by a mixture of independent and constrained refinement
Δρ_max_ = 1.03 e Å^−3^
Δρ_min_ = −0.87 e Å^−3^

### 

Data collection: *CrysAlis PRO* (Agilent, 2011 ▶); cell refinement: *CrysAlis PRO*; data reduction: *CrysAlis PRO*; program(s) used to solve structure: *SHELXS97* (Sheldrick, 2008 ▶); program(s) used to refine structure: *SHELXL97* (Sheldrick, 2008 ▶); molecular graphics: *X-SEED* (Barbour, 2001 ▶); software used to prepare material for publication: *publCIF* (Westrip, 2010 ▶).

## Supplementary Material

Crystal structure: contains datablock(s) global, I. DOI: 10.1107/S1600536812002693/xu5443sup1.cif

Structure factors: contains datablock(s) I. DOI: 10.1107/S1600536812002693/xu5443Isup2.hkl

Additional supplementary materials:  crystallographic information; 3D view; checkCIF report

## Figures and Tables

**Table 1:** Hydrogen-bond geometry (Å, °)

*D*—H⋯*A*	*D*—H	H⋯*A*	*D*⋯*A*	*D*—H⋯*A*
O1*W*—H11⋯O1	0.83 (1)	2.02 (2)	2.802 (2)	156 (3)
O1*W*—H12⋯O3	0.84 (1)	1.93 (1)	2.758 (2)	171 (3)
O2*W*—H21⋯O6	0.83 (1)	1.87 (1)	2.696 (2)	174 (2)
O2*W*—H22⋯O8	0.84 (1)	1.85 (1)	2.678 (2)	170 (2)
O3*W*—H31⋯O7	0.84 (1)	1.93 (1)	2.738 (2)	163 (3)
O3*W*—H32⋯O10	0.84 (1)	1.93 (1)	2.758 (2)	168 (3)
O4*W*—H41⋯O2^i^	0.84 (1)	1.87 (1)	2.710 (2)	174 (2)
O4*W*—H42⋯O4^i^	0.84 (1)	1.86 (1)	2.700 (2)	179 (2)

Symmetry code: (i) 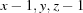.

**Fig. 1.**
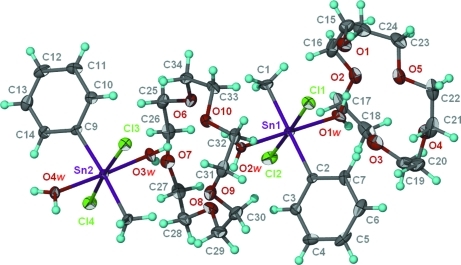
Thermal ellipsoid plot (Barbour, 2001) of the two independent molecules of MePhSnCl2(H2O)2.15-crown-5 at the 70% probability level; H atoms are drawn as spheres of arbitrary radius.

## supplementary crystallographic information

### Comment

Methylphenyltin dichloride in the form of its dihydrate furnishes a 2:1 co-crystal with 18-crown-6; the water molecule engages in double three-centre hydrogen bonding. The geometry at Sn is a distorted *trans*-C_2_SnO_4_ octahedron [C–Sn–C 147.8 (1) °], and the co-crystal exists as a dinuclear compound arising from an Sn···Cl bridge. The water molecule forms twin, bifurcated hydrogen bonds with the crown ether (Amini *et al.*, 1994). Replacing the 18-crown-6 molecule by the smaller 15-crown-5 molecule yields the corresponding diaqua 1:1 co-crystal (Scheme I). The six-coordinate organotin molecule and the crown ether of the two independent formula units (Fig. 1) are linked by O_water_···O_crown ether_ hydrogen bonds into a linear chain running along [101]. Each coordinated water molecule forms a pair of hydrogen bonds to the same crown ether; for the crown ethers, only four of the five O atoms are themselves engaged in hydrogen-bonding interactions (Table 1). The metal centres show *trans*-C_2_SnCl_2_OC_2_SnO_4_ octahedral coordination [C–Sn–C 175.3 (1), 178.9 (1) °].

### Experimental

Methylphenyltin dichloride was synthesized as reported (Amini *et al.*, 1994). The compound (0.28 g, 0.1 mmol) and 15-crown-5 (0.24 g, 0.1 mmol) were dissolved in chloroform (20 ml) to give a clear solution. The solution was set aside for the growth of colourless crystals.

### Refinement

Carbon-bound H-atoms were placed in calculated positions [C–H 0.95 to 0.99 Å, *U*_iso_(H) 1.2 to 1.5*U*_eq_(C)] and were included in the refinement in the riding model approximation. The water H-atoms were located in a difference Fourier map, and were refined with distance restraints of O—H 0.84 (1) and H···H 1.37 (1) Å; their temperature factors were refined. The final difference Fourier map had a peak at 0.70 Å from Sn1 and a hole at 0.85 Å from this atom.

### Crystal data

**Table:**

[Sn(CH_3_)(C_6_H_5_)Cl_2_(H_2_O)_2_]·C_10_H_20_O_5_	*F*(000) = 2192
*M**_r_* = 538.02	*D*_x_ = 1.591 Mg m^−^^3^
Monoclinic, *P*2_1_/*c*	Mo *K*α radiation, λ = 0.71073 Å
Hall symbol: -P 2ybc	Cell parameters from 20094 reflections
*a* = 16.4338 (3) Å	θ = 2.3–27.5°
*b* = 21.2112 (4) Å	µ = 1.41 mm^−^^1^
*c* = 14.0347 (3) Å	*T* = 100 K
β = 113.325 (2)°	Block, colourless
*V* = 4492.40 (15) Å^3^	0.30 × 0.25 × 0.20 mm
*Z* = 8	

### Data collection

**Table:**

Agilent SuperNova Dual diffractometer with Atlas detector	10200 independent reflections
Radiation source: SuperNova (Mo) X-ray Source	8806 reflections with *I* > 2σ(*I*)
Mirror	*R*_int_ = 0.038
Detector resolution: 10.4041 pixels mm^-1^	θ_max_ = 27.6°, θ_min_ = 2.4°
ω scans	*h* = −16→20
Absorption correction: multi-scan (CrysAlis PRO; Agilent, 2011)	*k* = −25→27
*T*_min_ = 0.677, *T*_max_ = 0.766	*l* = −16→18
39686 measured reflections	

### Refinement

**Table:**

Refinement on *F*^2^	Primary atom site location: structure-invariant direct methods
Least-squares matrix: full	Secondary atom site location: difference Fourier map
*R*[*F*^2^ > 2σ(*F*^2^)] = 0.028	Hydrogen site location: inferred from neighbouring sites
*wR*(*F*^2^) = 0.078	H atoms treated by a mixture of independent and constrained refinement
*S* = 1.05	*w* = 1/[σ^2^(*F*_o_^2^) + (0.0378*P*)^2^ + 1.5734*P*] where *P* = (*F*_o_^2^ + 2*F*_c_^2^)/3
10200 reflections	(Δ/σ)_max_ = 0.001
521 parameters	Δρ_max_ = 1.03 e Å^−^^3^
12 restraints	Δρ_min_ = −0.87 e Å^−^^3^

### Fractional atomic coordinates and isotropic or equivalent isotropic displacement parameters (Å^2^)

**Table:**

	*x*	*y*	*z*	*U*_iso_*/*U*_eq_	
Sn1	0.497819 (8)	0.365298 (7)	0.732756 (11)	0.01219 (5)	
Sn2	−0.004021 (8)	0.390877 (6)	0.251186 (10)	0.01131 (5)	
Cl1	0.42093 (4)	0.36257 (2)	0.85880 (4)	0.01982 (12)	
Cl2	0.57532 (4)	0.37216 (3)	0.60749 (5)	0.02279 (12)	
Cl3	−0.07321 (4)	0.38957 (2)	0.38435 (4)	0.01817 (11)	
Cl4	0.08337 (4)	0.38994 (2)	0.13603 (4)	0.02100 (12)	
O1	0.64624 (10)	0.47180 (7)	0.99113 (12)	0.0245 (3)	
O2	0.79243 (10)	0.45241 (7)	0.93383 (12)	0.0248 (4)	
O3	0.79425 (10)	0.32445 (8)	0.88931 (13)	0.0288 (4)	
O4	0.78284 (10)	0.27282 (7)	1.06645 (13)	0.0285 (4)	
O5	0.66865 (10)	0.35950 (7)	1.10291 (12)	0.0248 (4)	
O6	0.28218 (9)	0.47625 (7)	0.54533 (11)	0.0186 (3)	
O7	0.28947 (10)	0.41456 (7)	0.37626 (12)	0.0215 (3)	
O8	0.29535 (10)	0.28912 (7)	0.43429 (12)	0.0234 (3)	
O9	0.15323 (10)	0.27230 (7)	0.49947 (12)	0.0231 (3)	
O10	0.16993 (10)	0.39283 (7)	0.59396 (11)	0.0196 (3)	
O1W	0.62835 (10)	0.36383 (8)	0.86948 (13)	0.0216 (4)	
H11	0.6391 (18)	0.3880 (13)	0.9198 (17)	0.066 (11)*	
H12	0.6760 (12)	0.3496 (14)	0.870 (2)	0.076 (12)*	
O2W	0.36486 (10)	0.36413 (7)	0.60055 (12)	0.0158 (3)	
H21	0.3358 (14)	0.3972 (6)	0.5818 (17)	0.030 (7)*	
H22	0.3493 (16)	0.3383 (9)	0.5515 (14)	0.045 (9)*	
O3W	0.12643 (10)	0.39959 (7)	0.38318 (11)	0.0155 (3)	
H31	0.1741 (11)	0.3969 (14)	0.3750 (19)	0.052 (10)*	
H32	0.1359 (17)	0.3923 (14)	0.4454 (10)	0.054 (10)*	
O4W	−0.13670 (10)	0.38633 (7)	0.11331 (12)	0.0169 (3)	
H41	−0.1566 (16)	0.4090 (9)	0.0601 (13)	0.041 (8)*	
H42	−0.1619 (16)	0.3509 (6)	0.0982 (19)	0.046 (9)*	
C1	0.49268 (13)	0.46505 (10)	0.73747 (17)	0.0190 (5)	
H1A	0.4307	0.4788	0.7119	0.029*	
H1B	0.5207	0.4830	0.6936	0.029*	
H1C	0.5242	0.4795	0.8091	0.029*	
C2	0.50113 (12)	0.26551 (10)	0.72924 (15)	0.0125 (4)	
C3	0.47984 (13)	0.23275 (10)	0.63656 (16)	0.0174 (4)	
H3	0.4653	0.2553	0.5735	0.021*	
C4	0.47970 (15)	0.16718 (10)	0.63566 (19)	0.0236 (5)	
H4	0.4629	0.1451	0.5719	0.028*	
C5	0.50408 (15)	0.13422 (11)	0.7280 (2)	0.0262 (6)	
H5	0.5046	0.0894	0.7276	0.031*	
C6	0.52765 (15)	0.16639 (11)	0.82096 (19)	0.0251 (5)	
H6	0.5457	0.1437	0.8844	0.030*	
C7	0.52494 (14)	0.23173 (11)	0.82159 (17)	0.0201 (5)	
H7	0.5394	0.2536	0.8853	0.024*	
C8	−0.00427 (13)	0.29146 (10)	0.25344 (16)	0.0176 (5)	
H8A	−0.0636	0.2763	0.2429	0.026*	
H8B	0.0387	0.2765	0.3206	0.026*	
H8C	0.0120	0.2754	0.1979	0.026*	
C9	−0.01432 (13)	0.49079 (10)	0.23920 (15)	0.0131 (4)	
C10	0.00515 (13)	0.52678 (10)	0.32887 (17)	0.0179 (4)	
H10	0.0207	0.5064	0.3940	0.021*	
C11	0.00193 (14)	0.59220 (10)	0.32342 (18)	0.0222 (5)	
H11A	0.0170	0.6164	0.3850	0.027*	
C12	−0.02339 (16)	0.62219 (11)	0.2279 (2)	0.0243 (5)	
H12A	−0.0264	0.6669	0.2239	0.029*	
C13	−0.04426 (14)	0.58655 (10)	0.13845 (18)	0.0218 (5)	
H13	−0.0629	0.6069	0.0729	0.026*	
C14	−0.03793 (13)	0.52129 (10)	0.14445 (16)	0.0169 (4)	
H14	−0.0499	0.4973	0.0832	0.020*	
C15	0.71481 (15)	0.51687 (11)	1.0080 (2)	0.0274 (5)	
H15A	0.7645	0.5089	1.0755	0.033*	
H15B	0.6921	0.5600	1.0091	0.033*	
C16	0.74600 (16)	0.51079 (11)	0.9213 (2)	0.0305 (6)	
H16A	0.6947	0.5116	0.8535	0.037*	
H16B	0.7857	0.5463	0.9234	0.037*	
C17	0.80363 (16)	0.43121 (13)	0.84247 (18)	0.0322 (6)	
H17A	0.8394	0.4620	0.8227	0.039*	
H17B	0.7452	0.4269	0.7839	0.039*	
C18	0.84970 (16)	0.36874 (13)	0.8667 (2)	0.0340 (6)	
H18A	0.8617	0.3540	0.8066	0.041*	
H18B	0.9070	0.3728	0.9271	0.041*	
C19	0.83960 (16)	0.26769 (12)	0.9361 (2)	0.0356 (6)	
H19A	0.8990	0.2778	0.9895	0.043*	
H19B	0.8472	0.2404	0.8830	0.043*	
C20	0.78511 (17)	0.23446 (12)	0.9847 (2)	0.0359 (7)	
H20A	0.7242	0.2274	0.9324	0.043*	
H20B	0.8117	0.1930	1.0122	0.043*	
C21	0.72020 (16)	0.25463 (12)	1.1085 (2)	0.0338 (6)	
H21A	0.7415	0.2168	1.1528	0.041*	
H21B	0.6625	0.2445	1.0519	0.041*	
C22	0.70995 (16)	0.30867 (12)	1.17159 (19)	0.0313 (6)	
H22A	0.6731	0.2958	1.2095	0.038*	
H22B	0.7687	0.3220	1.2229	0.038*	
C23	0.67644 (16)	0.41776 (12)	1.15442 (18)	0.0277 (5)	
H23A	0.7390	0.4319	1.1835	0.033*	
H23B	0.6565	0.4134	1.2120	0.033*	
C24	0.61977 (16)	0.46448 (12)	1.07668 (19)	0.0287 (5)	
H24A	0.5573	0.4504	1.0501	0.034*	
H24B	0.6237	0.5058	1.1111	0.034*	
C25	0.28290 (15)	0.51087 (10)	0.45839 (18)	0.0221 (5)	
H25A	0.3110	0.5526	0.4809	0.026*	
H25B	0.2216	0.5174	0.4068	0.026*	
C26	0.33506 (15)	0.47263 (11)	0.41181 (18)	0.0232 (5)	
H26A	0.3402	0.4958	0.3532	0.028*	
H26B	0.3955	0.4645	0.4645	0.028*	
C27	0.34014 (16)	0.36933 (11)	0.34814 (19)	0.0248 (5)	
H27A	0.4011	0.3673	0.4024	0.030*	
H27B	0.3438	0.3817	0.2819	0.030*	
C28	0.29609 (16)	0.30617 (12)	0.33660 (17)	0.0252 (5)	
H28A	0.2347	0.3084	0.2834	0.030*	
H28B	0.3290	0.2743	0.3145	0.030*	
C29	0.25380 (15)	0.23016 (11)	0.43498 (19)	0.0262 (5)	
H29A	0.2958	0.1952	0.4425	0.031*	
H29B	0.2017	0.2244	0.3689	0.031*	
C30	0.22527 (15)	0.22969 (11)	0.52449 (19)	0.0242 (5)	
H30A	0.2067	0.1868	0.5350	0.029*	
H30B	0.2748	0.2431	0.5890	0.029*	
C31	0.12735 (15)	0.28559 (11)	0.58305 (18)	0.0239 (5)	
H31A	0.1327	0.2466	0.6240	0.029*	
H31B	0.0643	0.2985	0.5544	0.029*	
C32	0.18207 (15)	0.33657 (11)	0.65367 (17)	0.0215 (5)	
H32A	0.1629	0.3433	0.7115	0.026*	
H32B	0.2454	0.3244	0.6832	0.026*	
C33	0.21056 (14)	0.44701 (11)	0.65545 (16)	0.0202 (5)	
H33A	0.2698	0.4358	0.7080	0.024*	
H33B	0.1738	0.4624	0.6920	0.024*	
C34	0.21908 (14)	0.49757 (10)	0.58484 (17)	0.0209 (5)	
H34A	0.1611	0.5052	0.5271	0.025*	
H34B	0.2394	0.5374	0.6237	0.025*	

### Atomic displacement parameters (Å^2^)

**Table:**

	*U*^11^	*U*^22^	*U*^33^	*U*^12^	*U*^13^	*U*^23^
Sn1	0.00939 (9)	0.01244 (9)	0.01434 (9)	−0.00024 (5)	0.00428 (6)	−0.00276 (5)
Sn2	0.00972 (9)	0.01073 (9)	0.01321 (8)	−0.00006 (5)	0.00424 (6)	0.00017 (5)
Cl1	0.0168 (3)	0.0265 (3)	0.0189 (3)	0.00063 (19)	0.0100 (2)	−0.0033 (2)
Cl2	0.0261 (3)	0.0206 (3)	0.0305 (3)	−0.0015 (2)	0.0206 (3)	0.0001 (2)
Cl3	0.0180 (3)	0.0204 (3)	0.0202 (3)	0.00022 (19)	0.0120 (2)	0.0014 (2)
Cl4	0.0237 (3)	0.0234 (3)	0.0219 (3)	0.0028 (2)	0.0154 (2)	0.0009 (2)
O1	0.0195 (8)	0.0260 (9)	0.0259 (9)	−0.0047 (6)	0.0065 (7)	−0.0032 (7)
O2	0.0229 (9)	0.0294 (9)	0.0199 (8)	−0.0030 (7)	0.0062 (7)	0.0038 (7)
O3	0.0155 (8)	0.0380 (10)	0.0311 (10)	0.0011 (7)	0.0073 (7)	−0.0073 (8)
O4	0.0208 (9)	0.0226 (9)	0.0363 (10)	−0.0066 (7)	0.0050 (7)	−0.0039 (7)
O5	0.0208 (9)	0.0308 (10)	0.0199 (9)	−0.0019 (6)	0.0049 (7)	0.0017 (7)
O6	0.0158 (8)	0.0194 (8)	0.0208 (8)	0.0035 (6)	0.0072 (6)	0.0023 (6)
O7	0.0158 (8)	0.0250 (8)	0.0256 (9)	−0.0002 (6)	0.0102 (7)	−0.0016 (7)
O8	0.0257 (9)	0.0244 (9)	0.0193 (8)	−0.0038 (7)	0.0082 (7)	−0.0053 (7)
O9	0.0206 (8)	0.0243 (8)	0.0227 (8)	0.0042 (6)	0.0068 (7)	0.0039 (7)
O10	0.0199 (8)	0.0226 (8)	0.0154 (8)	−0.0005 (6)	0.0059 (6)	−0.0001 (6)
O1W	0.0111 (8)	0.0309 (10)	0.0195 (9)	0.0005 (6)	0.0026 (6)	−0.0115 (7)
O2W	0.0144 (8)	0.0141 (8)	0.0148 (8)	0.0019 (6)	0.0013 (6)	−0.0019 (6)
O3W	0.0091 (8)	0.0235 (8)	0.0134 (8)	−0.0011 (6)	0.0041 (6)	0.0008 (6)
O4W	0.0142 (8)	0.0154 (8)	0.0170 (8)	−0.0018 (6)	0.0018 (6)	0.0017 (6)
C1	0.0163 (11)	0.0133 (11)	0.0248 (12)	−0.0003 (8)	0.0053 (9)	−0.0044 (9)
C2	0.0086 (10)	0.0129 (10)	0.0172 (10)	0.0014 (7)	0.0062 (8)	−0.0008 (8)
C3	0.0148 (11)	0.0194 (11)	0.0176 (11)	0.0017 (8)	0.0060 (8)	−0.0003 (9)
C4	0.0211 (12)	0.0192 (11)	0.0310 (13)	−0.0016 (9)	0.0109 (10)	−0.0078 (10)
C5	0.0241 (14)	0.0136 (12)	0.0475 (16)	0.0047 (8)	0.0211 (12)	0.0058 (10)
C6	0.0207 (12)	0.0250 (12)	0.0349 (14)	0.0064 (9)	0.0168 (10)	0.0144 (11)
C7	0.0165 (11)	0.0268 (12)	0.0186 (11)	0.0033 (9)	0.0086 (9)	0.0044 (9)
C8	0.0183 (12)	0.0120 (11)	0.0196 (11)	0.0015 (7)	0.0046 (9)	0.0015 (8)
C9	0.0094 (10)	0.0120 (10)	0.0184 (11)	−0.0002 (7)	0.0061 (8)	−0.0005 (8)
C10	0.0129 (11)	0.0194 (11)	0.0210 (11)	−0.0006 (8)	0.0065 (8)	−0.0008 (9)
C11	0.0210 (12)	0.0205 (11)	0.0279 (13)	−0.0045 (9)	0.0125 (10)	−0.0078 (10)
C12	0.0231 (12)	0.0152 (11)	0.0393 (14)	−0.0002 (9)	0.0173 (11)	0.0002 (10)
C13	0.0177 (12)	0.0213 (12)	0.0268 (12)	0.0006 (9)	0.0093 (9)	0.0073 (9)
C14	0.0120 (10)	0.0178 (11)	0.0200 (11)	−0.0013 (8)	0.0055 (8)	0.0000 (9)
C15	0.0215 (13)	0.0156 (11)	0.0380 (15)	−0.0032 (9)	0.0041 (10)	−0.0035 (10)
C16	0.0222 (13)	0.0255 (13)	0.0353 (14)	−0.0036 (10)	0.0024 (10)	0.0119 (11)
C17	0.0221 (13)	0.0560 (18)	0.0189 (12)	−0.0112 (12)	0.0083 (10)	0.0049 (12)
C18	0.0173 (13)	0.0619 (19)	0.0257 (14)	−0.0080 (12)	0.0117 (11)	−0.0104 (12)
C19	0.0209 (13)	0.0367 (15)	0.0394 (15)	0.0090 (11)	0.0015 (11)	−0.0195 (12)
C20	0.0244 (14)	0.0218 (13)	0.0453 (16)	0.0025 (10)	−0.0036 (11)	−0.0102 (12)
C21	0.0200 (13)	0.0284 (14)	0.0408 (16)	−0.0094 (10)	−0.0011 (11)	0.0137 (12)
C22	0.0190 (13)	0.0442 (16)	0.0269 (13)	−0.0063 (11)	0.0051 (10)	0.0142 (12)
C23	0.0219 (13)	0.0424 (15)	0.0205 (12)	−0.0050 (10)	0.0101 (10)	−0.0074 (11)
C24	0.0209 (13)	0.0358 (14)	0.0304 (13)	−0.0018 (10)	0.0113 (10)	−0.0111 (11)
C25	0.0215 (12)	0.0179 (11)	0.0250 (12)	−0.0014 (9)	0.0073 (9)	0.0057 (9)
C26	0.0182 (12)	0.0266 (12)	0.0241 (12)	−0.0052 (9)	0.0076 (9)	0.0056 (10)
C27	0.0194 (12)	0.0376 (14)	0.0211 (12)	0.0051 (10)	0.0118 (10)	0.0003 (10)
C28	0.0237 (13)	0.0349 (14)	0.0167 (11)	0.0065 (10)	0.0077 (9)	−0.0053 (10)
C29	0.0237 (13)	0.0205 (12)	0.0313 (13)	−0.0020 (9)	0.0075 (10)	−0.0086 (10)
C30	0.0205 (12)	0.0160 (11)	0.0321 (13)	0.0005 (9)	0.0062 (10)	0.0012 (10)
C31	0.0203 (12)	0.0253 (12)	0.0271 (13)	0.0010 (9)	0.0105 (10)	0.0088 (10)
C32	0.0206 (12)	0.0284 (13)	0.0180 (11)	0.0055 (9)	0.0103 (9)	0.0074 (10)
C33	0.0145 (11)	0.0292 (12)	0.0160 (11)	0.0007 (9)	0.0051 (8)	−0.0056 (9)
C34	0.0151 (11)	0.0207 (12)	0.0244 (12)	0.0041 (8)	0.0052 (9)	−0.0051 (9)

### Geometric parameters (Å, °)

**Table:**

Sn1—C2	2.118 (2)	C10—C11	1.389 (3)
Sn1—C1	2.120 (2)	C10—H10	0.9500
Sn1—O2W	2.2377 (15)	C11—C12	1.390 (3)
Sn1—O1W	2.2416 (16)	C11—H11A	0.9500
Sn1—Cl1	2.5495 (5)	C12—C13	1.387 (3)
Sn1—Cl2	2.5509 (5)	C12—H12A	0.9500
Sn2—C8	2.109 (2)	C13—C14	1.388 (3)
Sn2—C9	2.127 (2)	C13—H13	0.9500
Sn2—O3W	2.2174 (15)	C14—H14	0.9500
Sn2—O4W	2.2730 (15)	C15—C16	1.502 (4)
Sn2—Cl3	2.5435 (5)	C15—H15A	0.9900
Sn2—Cl4	2.5521 (5)	C15—H15B	0.9900
O1—C15	1.424 (3)	C16—H16A	0.9900
O1—C24	1.438 (3)	C16—H16B	0.9900
O2—C16	1.428 (3)	C17—C18	1.497 (4)
O2—C17	1.438 (3)	C17—H17A	0.9900
O3—C19	1.431 (3)	C17—H17B	0.9900
O3—C18	1.429 (3)	C18—H18A	0.9900
O4—C20	1.419 (3)	C18—H18B	0.9900
O4—C21	1.428 (3)	C19—C20	1.499 (4)
O5—C23	1.412 (3)	C19—H19A	0.9900
O5—C22	1.426 (3)	C19—H19B	0.9900
O6—C25	1.428 (3)	C20—H20A	0.9900
O6—C34	1.429 (2)	C20—H20B	0.9900
O7—C26	1.425 (3)	C21—C22	1.498 (4)
O7—C27	1.425 (3)	C21—H21A	0.9900
O8—C28	1.423 (3)	C21—H21B	0.9900
O8—C29	1.427 (3)	C22—H22A	0.9900
O9—C30	1.419 (3)	C22—H22B	0.9900
O9—C31	1.425 (3)	C23—C24	1.495 (3)
O10—C32	1.426 (3)	C23—H23A	0.9900
O10—C33	1.434 (3)	C23—H23B	0.9900
O1W—H11	0.833 (10)	C24—H24A	0.9900
O1W—H12	0.839 (10)	C24—H24B	0.9900
O2W—H21	0.831 (9)	C25—C26	1.505 (3)
O2W—H22	0.836 (9)	C25—H25A	0.9900
O3W—H31	0.837 (9)	C25—H25B	0.9900
O3W—H32	0.839 (9)	C26—H26A	0.9900
O4W—H41	0.839 (9)	C26—H26B	0.9900
O4W—H42	0.843 (9)	C27—C28	1.501 (3)
C1—H1A	0.9800	C27—H27A	0.9900
C1—H1B	0.9800	C27—H27B	0.9900
C1—H1C	0.9800	C28—H28A	0.9900
C2—C3	1.392 (3)	C28—H28B	0.9900
C2—C7	1.395 (3)	C29—C30	1.505 (3)
C3—C4	1.391 (3)	C29—H29A	0.9900
C3—H3	0.9500	C29—H29B	0.9900
C4—C5	1.385 (3)	C30—H30A	0.9900
C4—H4	0.9500	C30—H30B	0.9900
C5—C6	1.384 (3)	C31—C32	1.501 (3)
C5—H5	0.9500	C31—H31A	0.9900
C6—C7	1.387 (3)	C31—H31B	0.9900
C6—H6	0.9500	C32—H32A	0.9900
C7—H7	0.9500	C32—H32B	0.9900
C8—H8A	0.9800	C33—C34	1.504 (3)
C8—H8B	0.9800	C33—H33A	0.9900
C8—H8C	0.9800	C33—H33B	0.9900
C9—C14	1.389 (3)	C34—H34A	0.9900
C9—C10	1.397 (3)	C34—H34B	0.9900
			
C2—Sn1—C1	178.92 (8)	C15—C16—H16B	110.1
C2—Sn1—O2W	89.73 (6)	H16A—C16—H16B	108.4
C1—Sn1—O2W	89.95 (7)	O2—C17—C18	107.84 (19)
C2—Sn1—O1W	88.93 (7)	O2—C17—H17A	110.1
C1—Sn1—O1W	91.35 (7)	C18—C17—H17A	110.1
O2W—Sn1—O1W	177.35 (6)	O2—C17—H17B	110.1
C2—Sn1—Cl1	91.02 (5)	C18—C17—H17B	110.1
C1—Sn1—Cl1	87.95 (6)	H17A—C17—H17B	108.5
O2W—Sn1—Cl1	89.17 (4)	O3—C18—C17	108.82 (19)
O1W—Sn1—Cl1	88.56 (5)	O3—C18—H18A	109.9
C2—Sn1—Cl2	90.95 (5)	C17—C18—H18A	109.9
C1—Sn1—Cl2	90.08 (6)	O3—C18—H18B	109.9
O2W—Sn1—Cl2	91.10 (4)	C17—C18—H18B	109.9
O1W—Sn1—Cl2	91.20 (5)	H18A—C18—H18B	108.3
Cl1—Sn1—Cl2	178.012 (18)	O3—C19—C20	107.94 (19)
C8—Sn2—C9	175.34 (8)	O3—C19—H19A	110.1
C8—Sn2—O3W	94.46 (7)	C20—C19—H19A	110.1
C9—Sn2—O3W	90.19 (7)	O3—C19—H19B	110.1
C8—Sn2—O4W	87.91 (7)	C20—C19—H19B	110.1
C9—Sn2—O4W	87.45 (7)	H19A—C19—H19B	108.4
O3W—Sn2—O4W	177.39 (5)	O4—C20—C19	108.1 (2)
C8—Sn2—Cl3	88.51 (6)	O4—C20—H20A	110.1
C9—Sn2—Cl3	91.39 (5)	C19—C20—H20A	110.1
O3W—Sn2—Cl3	87.29 (4)	O4—C20—H20B	110.1
O4W—Sn2—Cl3	93.89 (4)	C19—C20—H20B	110.1
C8—Sn2—Cl4	90.38 (6)	H20A—C20—H20B	108.4
C9—Sn2—Cl4	90.27 (5)	O4—C21—C22	107.80 (19)
O3W—Sn2—Cl4	85.95 (4)	O4—C21—H21A	110.1
O4W—Sn2—Cl4	92.93 (4)	C22—C21—H21A	110.1
Cl3—Sn2—Cl4	173.042 (18)	O4—C21—H21B	110.1
C15—O1—C24	114.49 (18)	C22—C21—H21B	110.1
C16—O2—C17	114.33 (18)	H21A—C21—H21B	108.5
C19—O3—C18	113.14 (19)	O5—C22—C21	108.3 (2)
C20—O4—C21	115.69 (19)	O5—C22—H22A	110.0
C23—O5—C22	113.15 (19)	C21—C22—H22A	110.0
C25—O6—C34	114.93 (16)	O5—C22—H22B	110.0
C26—O7—C27	113.27 (17)	C21—C22—H22B	110.0
C28—O8—C29	114.58 (17)	H22A—C22—H22B	108.4
C30—O9—C31	114.45 (17)	O5—C23—C24	107.61 (19)
C32—O10—C33	113.18 (17)	O5—C23—H23A	110.2
Sn1—O1W—H11	122 (2)	C24—C23—H23A	110.2
Sn1—O1W—H12	126 (2)	O5—C23—H23B	110.2
H11—O1W—H12	109.6 (16)	C24—C23—H23B	110.2
Sn1—O2W—H21	120.2 (16)	H23A—C23—H23B	108.5
Sn1—O2W—H22	124.8 (16)	O1—C24—C23	111.95 (18)
H21—O2W—H22	110.0 (15)	O1—C24—H24A	109.2
Sn2—O3W—H31	121.8 (18)	C23—C24—H24A	109.2
Sn2—O3W—H32	125.0 (18)	O1—C24—H24B	109.2
H31—O3W—H32	109.5 (15)	C23—C24—H24B	109.2
Sn2—O4W—H41	129.7 (16)	H24A—C24—H24B	107.9
Sn2—O4W—H42	117.5 (17)	O6—C25—C26	107.04 (17)
H41—O4W—H42	107.9 (15)	O6—C25—H25A	110.3
Sn1—C1—H1A	109.5	C26—C25—H25A	110.3
Sn1—C1—H1B	109.5	O6—C25—H25B	110.3
H1A—C1—H1B	109.5	C26—C25—H25B	110.3
Sn1—C1—H1C	109.5	H25A—C25—H25B	108.6
H1A—C1—H1C	109.5	O7—C26—C25	107.88 (17)
H1B—C1—H1C	109.5	O7—C26—H26A	110.1
C3—C2—C7	119.1 (2)	C25—C26—H26A	110.1
C3—C2—Sn1	121.43 (15)	O7—C26—H26B	110.1
C7—C2—Sn1	119.47 (15)	C25—C26—H26B	110.1
C2—C3—C4	120.4 (2)	H26A—C26—H26B	108.4
C2—C3—H3	119.8	O7—C27—C28	108.73 (18)
C4—C3—H3	119.8	O7—C27—H27A	109.9
C5—C4—C3	119.8 (2)	C28—C27—H27A	109.9
C5—C4—H4	120.1	O7—C27—H27B	109.9
C3—C4—H4	120.1	C28—C27—H27B	109.9
C6—C5—C4	120.2 (2)	H27A—C27—H27B	108.3
C6—C5—H5	119.9	O8—C28—C27	108.10 (18)
C4—C5—H5	119.9	O8—C28—H28A	110.1
C5—C6—C7	120.1 (2)	C27—C28—H28A	110.1
C5—C6—H6	120.0	O8—C28—H28B	110.1
C7—C6—H6	120.0	C27—C28—H28B	110.1
C6—C7—C2	120.4 (2)	H28A—C28—H28B	108.4
C6—C7—H7	119.8	O8—C29—C30	108.63 (18)
C2—C7—H7	119.8	O8—C29—H29A	110.0
Sn2—C8—H8A	109.5	C30—C29—H29A	110.0
Sn2—C8—H8B	109.5	O8—C29—H29B	110.0
H8A—C8—H8B	109.5	C30—C29—H29B	110.0
Sn2—C8—H8C	109.5	H29A—C29—H29B	108.3
H8A—C8—H8C	109.5	O9—C30—C29	107.13 (19)
H8B—C8—H8C	109.5	O9—C30—H30A	110.3
C14—C9—C10	119.1 (2)	C29—C30—H30A	110.3
C14—C9—Sn2	121.54 (15)	O9—C30—H30B	110.3
C10—C9—Sn2	119.39 (15)	C29—C30—H30B	110.3
C11—C10—C9	120.4 (2)	H30A—C30—H30B	108.5
C11—C10—H10	119.8	O9—C31—C32	112.95 (18)
C9—C10—H10	119.8	O9—C31—H31A	109.0
C10—C11—C12	120.0 (2)	C32—C31—H31A	109.0
C10—C11—H11A	120.0	O9—C31—H31B	109.0
C12—C11—H11A	120.0	C32—C31—H31B	109.0
C13—C12—C11	119.7 (2)	H31A—C31—H31B	107.8
C13—C12—H12A	120.1	O10—C32—C31	107.87 (17)
C11—C12—H12A	120.1	O10—C32—H32A	110.1
C14—C13—C12	120.2 (2)	C31—C32—H32A	110.1
C14—C13—H13	119.9	O10—C32—H32B	110.1
C12—C13—H13	119.9	C31—C32—H32B	110.1
C13—C14—C9	120.6 (2)	H32A—C32—H32B	108.4
C13—C14—H14	119.7	O10—C33—C34	108.52 (17)
C9—C14—H14	119.7	O10—C33—H33A	110.0
O1—C15—C16	108.00 (19)	C34—C33—H33A	110.0
O1—C15—H15A	110.1	O10—C33—H33B	110.0
C16—C15—H15A	110.1	C34—C33—H33B	110.0
O1—C15—H15B	110.1	H33A—C33—H33B	108.4
C16—C15—H15B	110.1	O6—C34—C33	107.53 (17)
H15A—C15—H15B	108.4	O6—C34—H34A	110.2
O2—C16—C15	108.16 (19)	C33—C34—H34A	110.2
O2—C16—H16A	110.1	O6—C34—H34B	110.2
C15—C16—H16A	110.1	C33—C34—H34B	110.2
O2—C16—H16B	110.1	H34A—C34—H34B	108.5
			
O2W—Sn1—C2—C3	47.21 (16)	Sn2—C9—C14—C13	179.99 (15)
O1W—Sn1—C2—C3	−135.08 (16)	C24—O1—C15—C16	170.03 (18)
Cl1—Sn1—C2—C3	136.38 (15)	C17—O2—C16—C15	162.70 (18)
Cl2—Sn1—C2—C3	−43.89 (15)	O1—C15—C16—O2	−70.0 (2)
O2W—Sn1—C2—C7	−132.65 (15)	C16—O2—C17—C18	−177.70 (19)
O1W—Sn1—C2—C7	45.07 (15)	C19—O3—C18—C17	−166.6 (2)
Cl1—Sn1—C2—C7	−43.48 (15)	O2—C17—C18—O3	63.0 (2)
Cl2—Sn1—C2—C7	136.25 (15)	C18—O3—C19—C20	163.1 (2)
C7—C2—C3—C4	1.9 (3)	C21—O4—C20—C19	168.37 (18)
Sn1—C2—C3—C4	−177.93 (15)	O3—C19—C20—O4	−65.0 (2)
C2—C3—C4—C5	−2.4 (3)	C20—O4—C21—C22	−165.08 (19)
C3—C4—C5—C6	0.7 (3)	C23—O5—C22—C21	−164.31 (18)
C4—C5—C6—C7	1.5 (3)	O4—C21—C22—O5	67.1 (2)
C5—C6—C7—C2	−2.0 (3)	C22—O5—C23—C24	−171.88 (18)
C3—C2—C7—C6	0.2 (3)	C15—O1—C24—C23	−86.3 (2)
Sn1—C2—C7—C6	−179.89 (15)	O5—C23—C24—O1	−59.4 (2)
O3W—Sn2—C9—C14	132.02 (16)	C34—O6—C25—C26	−166.02 (17)
O4W—Sn2—C9—C14	−46.85 (16)	C27—O7—C26—C25	−168.96 (18)
Cl3—Sn2—C9—C14	−140.69 (15)	O6—C25—C26—O7	62.8 (2)
Cl4—Sn2—C9—C14	46.07 (16)	C26—O7—C27—C28	166.69 (18)
O3W—Sn2—C9—C10	−46.41 (16)	C29—O8—C28—C27	178.94 (18)
O4W—Sn2—C9—C10	134.72 (16)	O7—C27—C28—O8	−61.8 (2)
Cl3—Sn2—C9—C10	40.88 (15)	C28—O8—C29—C30	−157.78 (18)
Cl4—Sn2—C9—C10	−132.36 (15)	C31—O9—C30—C29	−170.11 (17)
C14—C9—C10—C11	−0.8 (3)	O8—C29—C30—O9	70.7 (2)
Sn2—C9—C10—C11	177.71 (15)	C30—O9—C31—C32	83.9 (2)
C9—C10—C11—C12	2.0 (3)	C33—O10—C32—C31	173.00 (17)
C10—C11—C12—C13	−0.8 (3)	O9—C31—C32—O10	61.4 (2)
C11—C12—C13—C14	−1.5 (3)	C32—O10—C33—C34	162.44 (17)
C12—C13—C14—C9	2.7 (3)	C25—O6—C34—C33	165.49 (17)
C10—C9—C14—C13	−1.6 (3)	O10—C33—C34—O6	−67.5 (2)

### Hydrogen-bond geometry (Å, °)

**Table:**

*D*—H···*A*	*D*—H	H···*A*	*D*···*A*	*D*—H···*A*
O1W—H11···O1	0.83 (1)	2.02 (2)	2.802 (2)	156 (3)
O1W—H12···O3	0.84 (1)	1.93 (1)	2.758 (2)	171 (3)
O2W—H21···O6	0.83 (1)	1.87 (1)	2.696 (2)	174 (2)
O2W—H22···O8	0.84 (1)	1.85 (1)	2.678 (2)	170 (2)
O3W—H31···O7	0.84 (1)	1.93 (1)	2.738 (2)	163 (3)
O3W—H32···O10	0.84 (1)	1.93 (1)	2.758 (2)	168 (3)
O4W—H41···O2^i^	0.84 (1)	1.87 (1)	2.710 (2)	174 (2)
O4W—H42···O4^i^	0.84 (1)	1.86 (1)	2.700 (2)	179 (2)

Symmetry codes: (i) *x*−1, *y*, *z*−1.
